# Supplementary material for: Parasitization of Aphis gossypii Glover by Binodoxys communis Gahan Causes Shifts in the Ovarian Bacterial Microbiota
Source: Insects. 2023 Mar 25;14(4):314. doi: 10.3390/insects14040314 (PMC10142764; doi:10.3390/insects14040314)
Supplement: Supplementary file 1 [file insects-14-00314-s001.zip › insects-2246520-supplementary.pdf]

Supplementary Information

Parasitization of *Aphis gossypii* Glover by *Binodoxys communis Gahan* causes shifts in ovarian bacterial microbiota

Jinming Li [1,2,a], Zhe An [1,2,a], Junyu Luo [1,2], Xiangzhen Zhu [1,2], Li Wang [1,2], Kaixin Zhang [1,2], Dongyang Li [1,2], Jichao Ji [1,2], Lin Niu[1,2], Xueke Gao [1,2*] and Jinjie Cui [1,2*]

(Zhengzhou Research Base, State Key Laboratory of Cotton Biology, School of Agricultural Sciences, Zhengzhou University, Zhengzhou 450001, Henan, China)

This Supplementary Information contains:

Figure S1: Rarefaction curves based on species abundance data.

Table S1: Primers used for core endosymbiotic bacteria in *Aphis gossypii* by RT-qPCR.

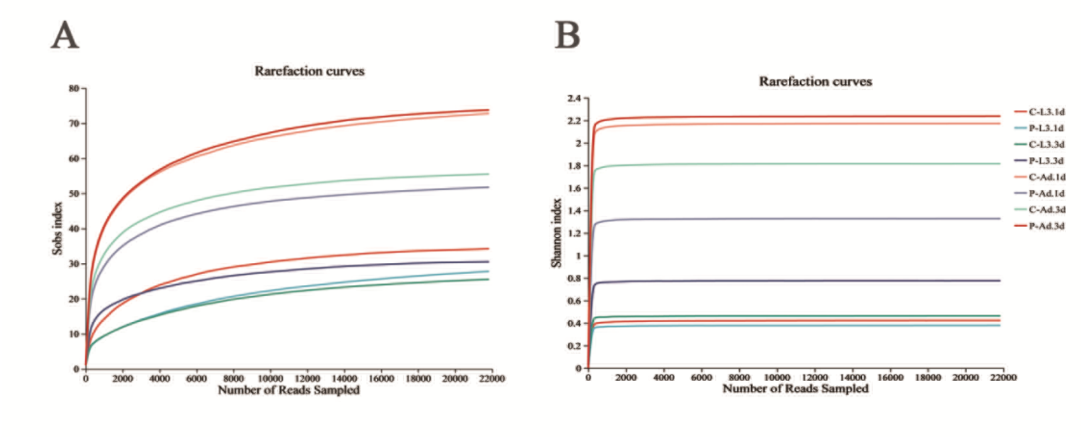

Figure S1. Rarefaction curves based on species abundance data. (A) The sample rarefaction curve (B) the Shannon index rarefaction curve. C-L3.1 d/P-L3.1d = control / parasitized aphids at third-instar nymph, 1 d, C-L3.3 d/P-L3.3 d = control / parasitized aphids at third-instar nymph, 3 d, C-Ad.1 d/P-Ad.1 d = control / parasitized aphids at adult, 1 d, C-Ad.3 d/P-Ad.3 d = control / parasitized aphids at adult, 3 d.

Table S1. **Primers used for core endosymbiotic bacteria in *Aphis gossypii* by RT-qPCR.**

S No	Name	Primer sequence (5'-3')
1	*Buchnera*	F: AGTATCGTAGAGGGAGGTA<br>R: CTTTCGCCACAGGTATTC
2	*Arsenophonus*	F: CATTCAAGACTGGTTAGC<br>R: TATTCCTCCACATCTCTAC
3	*Serratia*	F: GGGTTGTAAAGCACTTTCA<br>R: CTTCTTCTGCGAGTAACG
